# Tissue Reactivity of the 14F7 Mab Raised against N-Glycolyl GM3 Ganglioside in Tumors of Neuroectodermal, Mesodermal, and Epithelial Origin

**DOI:** 10.1155/2013/602417

**Published:** 2013-01-09

**Authors:** Rancés Blanco, Yisel Quintana, Damián Blanco, Mercedes Cedeño, Charles E. Rengifo, Milagros Frómeta, Martha Ríos, Enrique Rengifo, Adriana Carr

**Affiliations:** ^1^Department of Quality Control, Center of Molecular Immunology, 216 Street and 15 Avenue Atabey, Playa, P.O. Box 16040, 11600 Havana, Cuba; ^2^Department of Cell Biology and Tissues Banking, National Institute of Oncology and Radiobiology, 29 and F Street Vedado, Plaza de la Revolución, 10400 Havana, Cuba; ^3^Department of Pathology, Manuel Fajardo General Hospital, Zapata and D Street Vedado, Plaza de la Revolución, 10400 Havana, Cuba; ^4^Department of Neurosurgery, Juan Manuel Marquez Pediatric Hospital, Marianao, 11400 Havana, Cuba; ^5^Research and Development Direction, Center of Molecular Immunology, 216 Street and 15 Avenue Atabey, Playa, P.O. Box 16040, 11600 Havana, Cuba

## Abstract

The expression of N-glycolylneuraminic acid forming the structure of gangliosides and/or other glycoconjugates (Hanganutziu-Deicher antigen) in human has been considered as a tumor-associated antigen. Specifically, some reports of 14F7 Mab (a highly specific Mab raised against N-glycolyl GM3 ganglioside) reactivity in human tumors have been recently published. Nevertheless, tumors of epithelial origin have been mostly evaluated. The goal of the present paper was to evaluate the immunohistochemical recognition of 14F7 Mab in different human tumors of neuroectodermal, mesodermal, and epithelial origins using an immunoperoxidase staining method. Samples of fetal, normal, and reactive astrocytosis of the brain were also included in the study. In general, nontumoral tissues, as well as, low-grade brain tumors showed no or a limited immunoreaction with 14F7 Mab. Nevertheless, high-grade astrocytomas (III-IV) and neuroblastomas, as well as, sarcomas and thyroid carcinomas were mostly reactive with 14F7. No reaction was evidenced in medulloblastomas and ependymoblastomas. Our data suggest that the expression of N-glycolyl GM3 ganglioside could be related to the aggressive behavior of malignant cells, without depending on the tumor origin. Our data could also support the possible use of N-glycolyl GM3 as a target for both active and passive immunotherapies of malignancies expressing this molecule.

## 1. Introduction

Changes in the composition of cell surface glycolipids that take place during malignant transformation have been extensively described [1]. Particularly, numerous studies on glycolipids have been focused on gangliosides [2]. Gangliosides are glycosphingolipids containing sialic acid engaged in a wide variety of biological events that occur at vertebrate's cell membrane. They are widely distributed in both normal and tumoral human tissues of neuroectodermal origin [3, 4]. 

The most abundant sialic acid variants in mammals are N-acetylneuraminic acid (NeuAc) and N-glycolylneuraminic acid (NeuGc). NeuAc acid is the predominant sialic acid species expressed in mammalian brain gangliosides. Whereas, NeuGc is a predominant sialic acid species expressed in gangliosides from nonneural tissues of most nonhuman species [5, 6]. In contrast to NeuAc, the expression of the NeuGc forming the structure of gangliosides and/or other glycoconjugates (Hanganutziu-Deicher antigen) in human has been considered as a tumor-associated antigen [7]. The only structural difference between NeuAc and NeuGc is a single oxygen atom at the C-5 position of NeuGc catalyzed by the cytidine monophospho-N-acetylneuraminic acid hydroxylase (CMP-NeuAc hydroxylase) [8]. This minor difference is able to induce an immune response [9] as well as to develop specific antibodies raised against N-glycolylated gangliosides [10, 11].

The aberrant expression of the NeuGc residues in humans has been considered to be associated with the altered metabolism of malignant cells [9, 12, 13]. Normal human cells are incapable of synthesizing NeuGc due to a specific inactivating mutation in the CMP-NeuAc hydroxylase gene [14]. However, some authors have suggested an alternative pathway to the NeuGc synthesis from other intermediates of cellular metabolism in some human tumors [9].

Recently, some reports of 14F7 Mab (a highly specific Mab raised against N-glycolyl GM3 ganglioside) reactivity in formalin-fixed and paraffin-embedded tissues have been published. Nevertheless, epithelial-derived tumors have been mostly evaluated [15–19]. In this way, the analysis of NeuGcGM3 expression in different human neoplasms could be useful as a better basis for understanding the molecular pathogenesis of malignancies as well as to extend the assessment of this molecule as target for cancer immunotherapy. For these reasons, in this work was evaluated the recognition of 14F7 Mab in a serie of neuroectodermal, mesodermal, and epithelial derived tumors. Samples of fetal, normal, and reactive astrocytosis were also included in the study. 

## 2. Materials and Methods

### 2.1. Monoclonal Antibody

We used the 14F7 Mab (IgG1) a highly specific anti-NeuGcGM3 ganglioside antibody. This Mab was generated by immunization of Balb/c mice with NeuGcGM3 hydrophobically conjugated with human very low-density lipoproteins (VLDLs) adjuvated with Complete Freud adjuvant (CFA). Afterward, 14F7 Mab was obtained by the hybridoma resulting in the fusion of spleen cells with mouse myeloma cell line P3X63Ag653 as described in [10].

### 2.2. Tissue Specimens

Routinely processed, formalin-fixed, and paraffin-embedded archival samples with diagnosis of fetal tissues (3), normal adult tissues (10), reactive astrocytosis of the brain (3), pediatric brain tumors (35), sarcomas (30), and thyroid carcinomas (25) as well as frozen adult tissues (84 normal and 11 tumoral) were received from the pathology departments of Ramón González Coro Gyneco-Obstetric Hospital, Juan Manuel Márquez Pediatric Hospital, the legal-medicine department at “Amalia Simoni” Provincial Hospital of Camagüey, the National Institute of Neurology and Neuropathology, and the National Institute of Oncology and Radiobiology. Fetal tissues were obtained from 19-week-old fetus aborted by Rivanol, and normal tissues were removed at autopsy of healthy persons having suffered clinical death or by conventional intraoperatory biopsy. All samples were used after obtaining an approved consent by the institutional ethical committees.

### 2.3. Previous Processing

For fresh samples, tissues were frozen in liquid nitrogen and stored at −70°C until sectioning. Then, five micrometres sections were obtained in a cryostat and slides were stored at −20°C until they were used. These sections were fixed in 4% paraformaldehyde during 20 minutes at room temperature. All samples were washed in tap water and rehydrated in distilled water for 10 minutes and TBS for 5 minutes. Slides were incubated with biotin-blocking system (X0560, Dako, Denmark A/S), according to manufacture instructions. Afterward, tissue samples were washed with TBS during 10 min. For formalin-fixed and paraffin-embedded tissues, five micrometer serial sections from each block were obtained, and the slides were processed as it was previously described [16].

### 2.4. Immunohistochemical Staining and Evaluation

The method previously described in[16] was used. Briefly, the samples were incubated with 14F7 Mab followed by a peroxidase avidin-biotin system. Negative controls were performed substituting primary antibody for washing buffer (TBS). Colonic adenocarcinoma [16] and a breast infiltrating carcinoma [10] were taken as positive control for both paraffin-embedded and frozen tissues, respectively. Enzymatic activity was visualized with a DAB (K3465, Dako, Denmark A/S) solution, and slides were counterstained with Mayer's Hematoxylin (S2020, Dako, Denmark A/S). Staining of both cell membrane and cytoplasm was considered as positive for 14F7 Mab. A semiquantitative scoring system was used to define levels of reactivity. According to the staining pattern, the tumor sample was assigned to 1 of 4 scores: 0, no staining; 1, weak staining; 2, moderate staining; and 3, strong staining of malignant cells. All microscopic analyses were performed by two different observers.

## 3. Results

### 3.1. A Limited Reaction of 14F7 Mab Was Detected in Nontumoral Tissues

No reaction was observed with the 14F7 Mab in fetal tissues, except for a weak-to-moderate reactivity in less than 25% of brain neurons in 1/3 cases (Table 1). No staining was evidenced neither in formalin-fixed and paraffin-embedded (0/10) nor frozen (1/84) normal tissues, except for a weak reaction of mucous cells from small intestine (1/3) (Table 2). Similar results were obtained from reactive astrocytosis of the brain (0/3).

### 3.2. 14F7 Mab Staining Was Mainly Found in Frozen Tumors with Astrocytic Differentiation

An intense homogeneous and finely granular pattern of recognition was detected in 2/2 anaplastic astrocytomas, 1/2 oligoastrocytic tumors, while the glioblastoma (0/1) showed no reaction with 14F7 Mab. The reaction was mainly located on the plasmatic membrane and in more than 50% of malignant astrocytes. No staining was observed in oligodendrogliomas (0/3) and meningiomas (0/3) (Table 3).

### 3.3. The Reaction with 14F7 Mab Was Also Evidenced in Formalin-Fixed and Paraffin-Embedded Tumors

**Figure 1:**
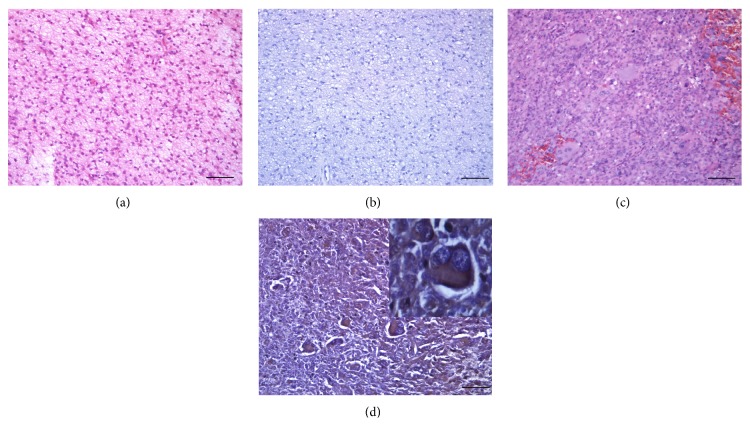
Hematoxylin and eosin staining of pilocytic astrocytoma (a) and glioblastoma (c). See no reaction of 14F7 Mab in pilocytic astrocytoma (b) while an intense reactivity with 14F7 Mab was observed in glioblastoma cells (d). The staining was located on both cell membrane and cytoplasm of malignant cells (inset on the upper-right corner, 400x magnification). Black bar = 100 *μ*m.

Pilocytic astrocytomas (0/4) were not recognized by 14F7 Mab. In contrast, 3/5 (60.00%), 5/8 (62.50%) of diffuse and anaplastic astrocytomas; and 2/2 of glioblastomas were moderate-to-intense reactive with 14F7 Mab in more than 50% of malignant cells (Table 4, Figure 1). One case of anaplastic astrocytoma and the oligodendroglioma showed a weak-to-moderate and finely granular reactivity mainly located in plasmatic membrane and also in the cytoplasm of less than 25% of malignant cells. No immunorecognition was observed in ependymomas, while 14F7 Mab was moderate-to-intense reactive at less than 25% of malignant cells in 1/5 anaplastic ependymomas. In the other hand, neuroblastomas (2/3) and ganglioneuroblastomas (2/2) exhibited a moderate-to-intense reaction in both the plasmatic membrane and the cytoplasm of more than 25% of malignant cells. Concerning to medulloblastomas (0/2) and ependymoblastomas (0/2) no immunostaining was evidenced.

### 3.4. Sarcomas Were Also Recognized by 14F7 Mab

The 14F7 Mab reactivity was detected in 25/30 (83.3%) of all studied soft tissue and bone sarcomas depending without the histopathological subtype. Considering the different histological subtypes of sarcomas, 8/8 muscular sarcomas, 3/3 vascular sarcomas, 2/2 peripheral nerve sheath tumours, 7/10 other fusocellular sarcomas, 2/3 liposarcomas, and 3/3 osteosarcomas were recognized by 14F7 Mab. No immunoreaction was evidenced in the synovial sarcoma (Table 5). 

**Figure 2:**
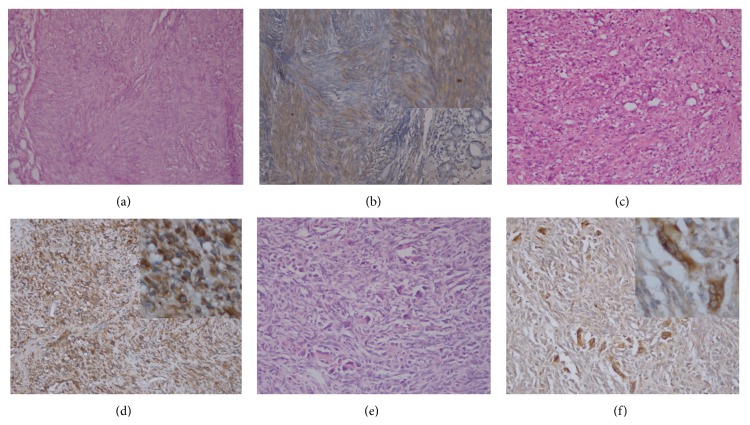
Hematoxylin and eosin staining of low-grade leiomyosarcoma of the stomach (a), malignant fibrous histiocytoma (c), and osteogenic sarcoma of the breast (e). Moderate reaction of 14F7 Mab in malignant smooth muscle cells. See no recognition of 14F7 in normal glands of the stomach (b). An intense immunostaining with 14F7 Mab located on both cell membrane and cytoplasm was detected in malignant fibrous histiocytoma (d) and osteoid cells (f) (inset on the upper-right corner, 400x magnification). Black bar = 100 *μ*m.

In general, the staining with 14F7 was observed as a finely granular reaction mainly located in the cell membrane but also in the cytoplasm of more than 50% of malignant cells (Figure 2). Almost all sarcomas were moderate-to-intense reactive with 14F7 although a weak intensity of staining was observed in a low-grade leiomyosarcoma.

### 3.5. Thyroid Carcinoma

**Figure 3:**
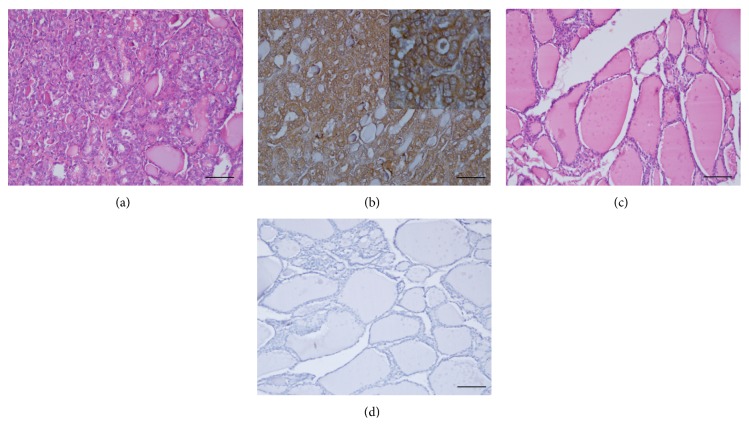
Hematoxylin and eosin staining of thyroid papillary carcinoma (a) and nontumoral adjacent area (c). Strong and finely granular reactivity of 14F7 Mab (b). The reaction was mainly located on the plasmatic membrane of malignant cells (inset on the upper-right corner, 400x magnification). Note no reaction of 14F7 in nontumoral thyroid tissue (d). Black bar = 100 *μ*m.

A moderate-to-intense reactivity in 23/25 (92.0%) of thyroid carcinoma was detected (Table 6). The reaction with 14F7 showed a finely granular pattern localized in both the plasmatic membrane and the cytoplasm of neoplastic cells (Figure 3).

## 4. Discussion

Unusual glycosylated or sialylated gangliosides have been identified with monoclonal antibodies generated against tumor-associated antigens, and they were considered as targets for use in passive and active immunotherapy of some malignant neoplasms [10, 11]. Between them, the expression of a nonhuman sialic acid (N-glycolylneuraminic) forming the structure of gangliosides and/or other glycoconjugates has been considered one of the most important antigens [1, 20]. 

The structural difference between N-acetylneuraminic (normal constituent of human tissues) and N-glycolylneuraminic (tumor-associated antigen) is crucial in many aspects of the cellular behavior [12, 21] and has permitted the development of specific antibodies raised against the Hanganutziu-Deicher (HD) antigen or N-glycolylated gangliosides as well as their immunohistochemical evaluation using both frozen and formalin-fixed and paraffin-embedded tissues [9, 22]. The antigenic determinant of HD antigen is N-glycolylneuraminic acid. Therefore, HD is classified as a heterophile antigen and chemically defined as a glycolipid and/or glycoprotein (glycoconjugates) which contains NeuGc. This antigen has been reported to be almost absent in normal human tissues but can be expressed on a variety of human malignant cells [23].

In our study, both frozen and formalin-fixed and paraffin-embedded nontumoral human tissues were not reactive with 14F7 Mab (IgG1 highly specific against N-glycolyl GM3 ganglioside), except for a weak-to-moderate staining of some neurons in fetal tissues. Additionally, we observed an intense staining of 14F7 in mucous cells from small intestine. In previous studies, normal eukaryotic cells were able to take in a portion of ingested NeuGc and process it for their own glycoconjugates [13, 20]. In line with this, small levels of expression of NeuGc have been found in some normal human tissues (e.g., epithelial cells and their secretions) [12]. The limited reactivity of 14F7 Mab in nontumoral tissues confirmed that 14F7 Mab is able to distinguish between the N-glycolyl and the N-acetyl functions of the GM3 ganglioside [10]. Furthermore, the limited recognition of 14F7 Mab in other normal tissues has been also reported by our group [16–19]. 

In contrast, our group reported the expression of NeuGcGM3 in breast tumors using both thin layer chromatography (TLC) immunostaining and fast atom bombardment mass spectroscopy (FAB/MS) analysis [24]. In addition, we published the immunohistochemical recognition of the 14F7 Mab in breast infiltrating ductal carcinoma and melanoma by immunohistochemistry using frozen tissues fixed in 4% paraformaldehyde. This finding suggested that the structure recognized in breast tumors could be the oligosaccharide core of NeuGcGM3 present in glycoconjugates [10]. The *in vivo* tissular expression of NeuGcGM3 was also confirmed by the radioimmunoscintigrafic technique using 14F7 Mab labelled with 99mTc [25]. Afterward, we reported the immunohistochemical reactivity of 14F7 in a variety of formalin-fixed and paraffin-embedded tumor tissues, despite the extraction of glycolipids during the routine histological procedures [16–19].

Recently, Scursoni et al. reported the recognition of 14F7 Mab in pediatric neuroblastoma using formalin-fixed and paraffin-embedded tissues and suggested that the expression of NeuGcGM3 ganglioside is preserved in the more aggressive tumors. Moreover, a clinical trial with Racotumomab (anti-idiotypic vaccine) in pediatric neuroectodermal tumors has been suggested in [26]. In this study, we describe the reactivity of the 14F7 in tumors of the central nervous system using both frozen tissues after 4% paraformaldehyde fixation and formalin-fixed and paraffin-embedded tissues. Malignancies with astrocytic differentiation and among them: diffuse and anaplastic astrocytomas, glioblastomas, an neuroblastomas were mainly recognized by 14F7 Mab. Our data seems to be also in agreement with the preferential reactivity of 14F7 Mab in more aggressive types of human astrocytoma. In line with this, the progression of malignant brain tumors has been associated with altered gangliosides composition and distribution [2].

On the other hand, a preliminary study about the reactivity of 14F7 in Ewing sarcoma has been previously described suggesting the potential use of NeuGcGM3 for cancer immunotherapy [26]. Here, we obtained the reactivity of 14F7 Mab in almost all soft and nonsoft tissues sarcomas without taking into account the histopathological classification. At present, a lot of studies are focused to better understand the molecular pathogenesis of sarcomas as well as the identification of reliable molecular markers and possible therapeutic targets. Some of these studies have been focused in sialic acid content [27]. Authors have reported increased amount of serum total sialic acid as well as the detection of N-glycolylneuraminic acid antibody in patient bearing sarcomas [28]. Interestingly, our group has evidence about the occurrence of higher levels of antibodies raised against NeuGcGM3 in patients bearing sarcomas (Carr A, unpublished data).

Neoplastic transformation of the thyroid gland has been reported to be accompanied by changes in cellular sialylation. A limited or absent expression of sialic acid in the surface of follicular cells in normal thyroid glands, adenomas, and goiters has been demonstrated. In contrast, a weak-to-intense positivity for sialic acid was found in thyroid carcinomas [29]. In this study, we reported the immunohistochemical recognition of 14F7 Mab in the majority of thyroid carcinomas but not in their normal counterpart. In this way, our data permit to consider the potential use of NeuGcGM3 as recognized by 14F7 in both the distinction of malignant from benign thyroid lesions and in being a potential target for active and passive immunotherapy in persistent and recurrent thyroid carcinomas.

Finally, intratumoral hypoxia (low oxygen tension) has been associated with aggressive disease, poor prognosis, and resistance to conventional therapies of malignant brain tumors, sarcomas, and thyroid carcinomas [30–32]. Tumor hypoxia has been considered responsible of NeuGcGM2 ganglioside expression in human cancer cells through the incorporation of NeuGc. The effect of hypoxia could be to expedite sialic acid transport from the external medium, in relation to the increment of sialin expression (a sialic acid molecule transporter) [33]. The role of NeuGcGM3 in tumoral progression as well as its suppressor properties has been previously reported [34, 35]. In this way, studies focused on the evaluation of intratumoral hypoxia and NeuGcGM3 relations are being planned in our lab.

## 5. Conclusions

The expression of NeuGcGM3 in neuroectodermal, mesodermal, and epithelial derived tumors but not in normal sections suggests that the expression of this ganglioside could be related to the aggressive behavior of malignant cells, without depending on the tumor origin. Our data could support the possible use of NeuGcGM3 as a target for both active and passive immunotherapies of malignancies expressing this molecule.

## Figures and Tables

**Table 1:** Immunorecognition of 14F7 Mab in formalin-fixed and paraffin-embedded nontumoral tissues.

Samples	Score		
	0	1	Total (%)
Central nervous system			
Fetal			
Neurons	2	1	1/3 (33.33)
Glias	3	0	0/3
Normal			
Brain	3∗	0	0/3
Cerebellum	4	0	0/4
Reactive astrocytosis	3	0	0/3
Normal thyroid	3	0	0/3

0: no staining; 1: weak staining; ∗samples from the normal tissue located in peritumoral area.

**Table 2:** Reactivity of 14F7 Mab in frozen normal tissues.

Samples	14F7 Mab reactivity	
	No. cases/total	Intensity of reaction
Skin	0/3	−
Gastrointestinal tract		
Esophagus	0/3	−
Estomach	0/3	−
Small intestine	1/3	+++
Large intestine	0/3	−
Liver	0/3	−
Pancreas	0/3	−
Respiratory tract		
Lung	0/4	−
Urogenital tract		
Testis	0/4	−
Prostate	0/3	−
Kidney	0/4	−
Urinary bladder	0/3	−
Ureter	0/3	−
Nervous system		
Brain	0/4	−
Cerebellum	0/4	−
Brain stem	0/3	−
Spinal cord	0/1	−
Peripheral nervous	0/1	−
Immune system		
Thymus	0/1	−
Tonsils	0/1	−
Lymph node	0/2	−
Spleen	0/3	−
Cardiovascular system		
Heart	0/3	−
Artery	0/4	−
Vein	0/4	−
Muscle		
Estriated	0/4	−
Smooth	0/4	−
Endocrine system		
Pituitary	0/2	−
Thyroid	0/1	−

(−): negative; (+++): strong staining of cells.

**Table 3:** Immunostaining of 14F7 Mab in frozen tumors from central nervous system.

Histopathological type^*^	Score		
	0	3	Total (%)
Anaplastic astrocytoma	0	2	2/2 (100)
Glioblastoma	1	0	0/1
Oligodendroglioma	3	0	0/3
Oligoastrocytoma	1	1	1/2 (50)
Meningioma	3	0	0/3

^*^WHO grade classification. 0: no reaction; 3: strong staining of malignant cells.

**Table 4:** Recognition of 14F7 Mab in primary nervous system tumors of pediatrics patients.

Histopathological type^*^			Score		
	0	1	2	3	Total (%)
Pilocytic astrocytoma	4	0	0	0	0/4
Diffuse astrocytoma	2	0	1	2	3/5 (60.00)
Anaplastic astrocytoma	3	1	1	3	5/8 (62.50)
Glioblastoma	0	0	0	2	2/2
Oligodendroglioma	0	1	0	0	1/1
Ependymoma	1	0	0	0	0/1
Anaplastic ependymoma	4	0	1	0	1/5 (20.00)
Medulloblastoma	2	0	0	0	0/2
Neuroblastoma	1	0	0	2	2/3 (66.66)
Ganglioneuroblastoma	0	0	2	0	2/2
Ependymoblastoma	2	0	0	0	0/2

^*^WHO grade classification. 0: no staining; 1: weak staining; 2: moderate staining; 3: strong staining of malignant cells.

**Table 5:** Immunoreactivity of 14F7 Mab in sarcomas.

Histopathological type			Score		
	0	1	2	3	No. cases
Soft tissue sarcoma					
Leiomyosarcoma	0	1	2	4	7/7
Rhabdomyosarcoma	0	0	0	1	1/1
Endoteliosarcoma	0	0	0	1	1/1
Hemangiopericytoma	0	0	0	2	2/2
Neurofibrosarcoma	0	0	0	1	1/1
Malignant schwannoma	0	0	0	1	1/1
Fibrosarcoma	2	0	0	1	1/3
Malignant fibrous histiocytoma	1	0	0	3	3/4
NOS (not otherwise specified)	0	0	0	3	3/3
Liposarcoma	1	0	0	2	2/3
Synovial sarcoma	1	0	0	0	0/1
Nonsoft tissue sarcoma					
Osteosarcoma	0	0	0	3	3/3

Total					25/30 (83.3%)

NOS: not otherwise specified. 0: no staining; 1: weak staining; 2: moderate staining; 3: strong staining of malignant cells.

**Table 6:** Staining of 14F7 Mab in thyroid carcinomas.

Histopathological type^*^		Score		
	0	2	3	No. cases (%)
Thyroid carcinoma				
Papillary	1	0	11	11/12
Follicular	1	1	9	10/11
Medullary	0	1	1	2/2

Total	2	2	21	23/25 (92.0%)

^*^WHO grade classification. 0: no reaction; 2: moderate staining; and 3: strong staining of malignant cells.
